# Experimental Cancer Cachexia Changes Neuron Numbers and Peptide Levels in the Intestine: Partial Protective Effects after Dietary Supplementation with L-Glutamine

**DOI:** 10.1371/journal.pone.0162998

**Published:** 2016-09-16

**Authors:** Geraldo E. Vicentini, Luciane Fracaro, Sara R. G. de Souza, Heber A. Martins, Flávia A. Guarnier, Jacqueline N. Zanoni

**Affiliations:** 1 Department of Morphological Sciences, Universidade Estadual de Maringa, Maringa, Parana, Brazil; 2 Department of General Pathology, Universidade Estadual de Londrina, Londrina, Parana, Brazil; University of California Los Angeles, UNITED STATES

## Abstract

Gastrointestinal dysmotility frequently occurs in cancer cachexia and may result from damage to enteric innervation caused by oxidative stress, especially due to glutathione depletion. We assessed the effect of dietary supplementation with 20 g/kg l-glutamine (a glutathione precursor) on the intrinsic innervation of the enteric nervous system in healthy and Walker 256 tumor-bearing Wistar rats during the development of experimental cachexia (14 days), in comparison with non-supplemented rats, by using immunohistochemical methods and Western blotting. The total neural population and cholinergic subpopulation densities in the myenteric plexus, as well as the total population and VIPergic subpopulation in the submucosal plexus of the jejunum and ileum, were reduced in cachectic rats, resulting in adaptive morphometric alterations and an increase in vasoactive intestinal peptide (VIP) and calcitonin gene-related peptide (CGRP) expression, suggesting a neuroplastic response. l-glutamine supplementation prevented decrease in myenteric neuronal density in the ileum, morphometric alterations in the neurons and nerve fibers (in both the plexuses of the jejunum and ileum), and the overexpression of VIP and CGRP. Cancer cachexia severely affected the intrinsic innervation of the jejunum and ileum to various degrees and this injury seems to be associated with adaptive neural plasticity. l-glutamine supplementation presented partial protective effects on the enteric innervation against cancer cachexia, possibly by attenuating oxidative stress.

## Introduction

Most cancer patients (50–80%) develop cachexia, a frequent comorbidity of cancer that accounts for over 20% of deaths in the terminal stage and represents one of the most prevalent public health problems worldwide [1]. Cancer cachexia is characterized by a hypermetabolic state, resulting in the loss of body mass, skeletal muscle mass, and, possibly, adipose tissue [1, 2]. The cachexia pathophysiology mechanism is complex, however, a central role has been assigned to activation of the immune system with an excessive production of pro-inflammatory cytokines, which in turn may increase reactive oxygen species (ROS) production [2, 3]. Thus, systemic inflammation effects have influenced the quality of life of the cancer patients [4]. Cancer-related cytokines activate multiple signaling pathways to promote increased production of ROS that, together with decreased antioxidant defenses, lead to a condition known as oxidative stress [1, 3, 5], which stimulates injury to critical cellular components, harming nucleic acids and membrane lipids, and modifying enzymes and other proteins, ultimately leading to severe cell damage [6, 7].

Cellular redox state is governed by the elevated ratio of reduced glutathione (GSH) to oxidized glutathione (GSSG), and as a result of ROS combat GSH may be greatly depleted [7]. Glutathione is the most important thiol-containing antioxidant in the intracellular environment, consisting of the amino acids glutamate, cysteine, and glycine [8]. Depletion of GSH as observed in several diseases, such as cancer, leads to cell damage, even in extratumoral tissues [7, 9]. Its main targets are cells with high oxidative metabolism, such as neurons, in which GSH depletion may result in neurodegeneration [8, 10].

In many cancer patients, the maintenance of gastrointestinal function is critical to the management of comorbidities, particularly cachexia [11]. Several studies reported digestive and motility disorders associated with cancer, such as anorexia, dysphagia, constipation, diarrhea, abdominal pain, and intestinal obstruction [12, 13]. In all of these disorders, the enteric nervous system (ENS) is compromised to some degree, which may lead to neuropathy [13, 14]. Among the neurons that comprise the ENS myenteric and submucosal plexuses, neurons that produce the neurotransmitter acetylcholine by the enzyme choline acetyltransferase (CHAT) are the most abundant [15]. These neurons comprise the cholinergic circuit, which mediates motility, mucosal secretory, intrinsic sensory and vascular reflexes [14] and may be affected by the systemic effects of cachexia development. It seems plausible that some factors produced by tumoral tissue can actively affect the ENS [16]. Some studies have reported ENS alterations in colon cancer and their effects on neural elements in areas affected by the tumor in comparison with the effects in distal areas [16–19]. However, information about the involvement of enteric innervation when the cancer is located in extra-intestinal tissues is sparse. Therefore, we propose firstly to investigate whether cancer-related cachexia development may induce morpho-quantitative changes in enteric innervation, including CHAT-containing neurons in the small intestine, since it comprises 2/3 of the total intestinal length and contains 90% of the organ’s total absorptive surface [20].

Neuroendocrine peptides, such as vasoactive intestinal polypeptide (VIP), and calcitonin gene related peptide (CGRP), have been investigated in several studies in colon carcinoma patients and colon cancer experimental models and have been shown to be altered in neurons and nerve fibers [18, 19, 21, 22]. VIP and CGRP have an important regulatory role in the gastrointestinal tract, influencing motility, blood flow mucosal secretions and the immune system [14]. CGRP plays a neuromodulatory role in sensory systems [14], being present in intrinsic and extrinsic innervation of the ENS [17, 23], while VIP, in addition to ENS functions, has exhibited antioxidant and anti-inflammatory properties [24, 25]. Therefore, a complementary investigation to this study evaluated the morphometry of VIP and CGRP-containing nerve fibers and morpho-quantitative changes in VIP-containing neurons of the small intestine in a cancer cachexia experimental model. Walker 256 tumor was the chosen model as it is appropriate for studying the physiopathological and morphological changes during cancer development [5, 26, 27]. In this model, subcutaneously implanted carcinomatous cells gradually generate an inflammatory and oxidative stress state at both the tissue and systemic levels [5, 27–29]. Regardless of the recent study on interstitial cells of Cajal (ICC) in the Walker 256 tumor-bearing rat model [30], no study to date has focused on the ENS in extra-intestinal cancer models.

Another aspect evaluated in this study is based on evidence that antioxidant glutathione is depleted because of oxidative stress in the neuronal tissue, which has been reported in the Walker 256 tumor model [27], and on evidence that supplementation with a glutathione precursor, l-glutamine, has produced neuroprotective effects on ENS [31–33]. Thus, we tested the effects of dietary l-glutamine supplementation on enteric innervation in Walker 256 tumor-bearing rats.

## Materials and Methods

### Ethics

All procedures described in this study were performed in agreement with international ethical guidelines and were previously approved by the Ethics Committee in Animal Experimentation (CEAE) of the Universidade Estadual de Maringá –Parana—Brazil (UEM), under the protocol number 099/2012.

### Animals

Thirty-two 57-day-old male Wistar rats (*Rattus norvegicus*, weighing 189–223 g) were obtained from the Central Animal Facility of the Universidade Estadual de Maringá. The animals were randomly assigned to four experimental groups with water and food available *ad libitum* (n = 8 per group): control (C); control supplemented with 2% l-glutamine (CG); Walker 256 tumor (TW); and Walker 256 tumor supplemented with 2% l-glutamine (TWG). All animals were housed in polypropylene cages measuring 40 cm × 33 cm × 17 cm (length, width, and height) and maintained at a controlled environmental temperature (23 ± 2°C) and lighting regime (12-h/12-h dark/light cycle) during the 14-day experimental period.

### Experimental Induction of Walker 256 Tumors

For experimental tumor induction in the TW and TWG group animals, Walker 256 carcinosarcoma cells were suspended in 16.5 mM phosphate-buffered saline (PBS), pH 7.5, and subcutaneously injected into the right flank of each animal. A total of 8 × 10^7^ viable cells (determined in a Neubauer chamber using the trypan blue dye exclusion method) were introduced in each animal. Animals belonging to the C and CG groups received PBS (pH 7.5) injections on the same day at the same anatomical site. Walker 256 carcinosarcoma is an extensively tested and standardized model for studying cancer and cachexia. All experimental procedures were performed starting on the 14th day after tumor cell inoculation. This period was chosen based on a 15-day survival time after Walker 256 tumor implantation [5, 26].

### Cancer and Cachexia Development

The cachectic state was determined by assessing the body weight at the end of the experimental period, the weight variation [34], and the cachexia index [5]. Weight variation was calculated as the difference between the final and initial total body weight for each tumor-bearing animal (TW and TWG group) minus the tumor mass. Food consumption was assessed every 2 days (g/day) and expressed as g/100 g body weight/day.

### l-Glutamine Supplementation

During the experimental period, animals in the C and TW groups (not supplemented) were fed with a Nuvital standard balanced diet (Nuvilab, Colombo, PR, Brazil). The diets of rats in the CG and TWG group comprised the ground standard diet supplemented with l-glutamine (Deg, São Paulo, SP, Brazil); pellets with a concentration of 2% l-glutamine (20 g/Kg) were prepared from this [25, 31, 35]. Supplementation began on the day of tumor cell inoculation.

### Tissue Harvesting and Processing

At the end of the experimental period, animals were fasted for 12 h before being weighed and euthanized under intraperitoneal anesthesia (40 mg/kg body weight thiopental—Abbott Laboratories, Chicago, IL, USA). Next, a laparotomy was performed and the entire small intestine was removed. The area was obtained by measuring the intestinal length immediately after each animal had been euthanized. The following segments were separated: the jejunum (10 cm segments taken from the midpoint of the small intestine) and the ileum (first 6 cm oral to the ileocecal junction). A sample of each segment was then opened along the mesenteric border and the diameter was measured using a millimeter ruler. The total area of each segment was calculated by diameter × length and expressed in cm^2^. The harvested tissue was then washed with 0.1 M PBS (pH 7.4) and filled with Zamboni’s fixative, followed by tying off the ends. The intestinal segments were incubated in fixative for 18 h at 4°C. These segments were opened along the mesenteric border and repeatedly washed in 80% ethanol to completely remove the fixative. The tissues were then dehydrated in an increasing ethanol series (95% and 100%), cleared with xylene, gradually rehydrated in a decreasing ethanol series (100%, 90%, 80%, and 50%), and stored in PBS containing 0.08% sodium azide (s2002; Sigma-Aldrich, Inc., St. Louis, MO, USA) at 4°C [36]. Fixed tissues were cut into small pieces measuring approximately 1 cm^2^ and microdissected under a Stemi DV4 stereo microscope (Zeiss, Jena, Germany) to obtain whole-mount preparations of the muscle and submucosal layers. The study of ENS nerve plexuses requires removal of the mucosal and submucosal layers to expose the myenteric plexus and removal of the muscle layers and peeling of the mucosal layer to expose the submucosal plexus.

### Myenteric and Submucosal Plexuses Immunohistochemistry

#### Double staining for HuC/D and ChAT proteins in myenteric neurons

Double staining for HuC/D and ChAT was performed to assess the general neuronal population (HuC/D—pan-neuronal marker protein) and the cholinergic (neurons expressing the marker enzyme—ChAT) subpopulation of the myenteric plexus. The whole-mount preparations were washed three times with 0.1 M PBS pH 7.4 + 1.5% Triton X-100 (T8532; Sigma-Aldrich, Inc.) for 10 min and incubated in the same solution containing 1% bovine serum albumin (BSA; Sigma-Aldrich, Inc.) and 10% normal donkey serum for 2 h at room temperature (RT) to block nonspecific binding. The tissues were then removed from this solution, incubated simultaneously with the primary antibodies (double staining) in blocking solution (48 h at 4°C), washed in PBS + 0.5% Triton X-100 (3 × 5 min), and incubated with the secondary antibody (2 h at RT) as described in Table 1. After this incubation, three washes were performed (5 min) with 0.1 M PBS (pH 7.4) and, finally, the whole-mount preparations were slide-mounted with Prolong Gold antifade (P36930, Molecular Probes, Eugene, OR, USA) and stored at 4°C.

**Table 1:** Characteristics of the primary and secondary antibodies used in immunoreactions.

Primary antibodies	Source	Immunohistochemical Dilution	Western blot Dilution
Monoclonal Mouse anti—HuC/D; A-21271	Molecular Probes, Invitrogen, USA	1:500	
Polyclonal Goat anti—choline Acetyltransferase (CHAT); AB1582*	Merck Millipore Corporation, USA;	1:200	1:1000
Polyclonal Rabbit anti—Vasoactive intestinal peptide (VIP); sc-20727*	Santa Cruz Biotechnolog, USA	1:500	1:500
Polyclonal Rabbit anti—Calcitonin gene-related peptide (CGRP); AB-15360*	Merck Millipore Corporation, USA	1:500	1:1000
**Secondary Antibodies**			
Alexa fluor 488 (Donkey anti-mouse); A-21202	Molecular Probes, Invitrogen, USA	1:500	
Alexa fluor 546 Donkey anti-goat; A-11035	Molecular Probes, Invitrogen, USA	1:500	
Alexa fluor 568 (Goat anti-rabbit); A-11036	Molecular Probes, Invitrogen, USA	1:500	
Goat anti-Rabbit -HRP conjugate; A-16104*	Novex, Invitrogen, USA		1:1000
Rabbit anti-Goat -HRP conjugate; A-16136*	Novex, Invitrogen, USA		1:2000

* Antibodies use in Western blot.

#### Double staining for HuC/D and VIP in submucosal neurons and single staining for myenteric VIP-containing nerve fiber

Submucosal and myenteric layer whole-mount preparations were washed with 0.1 M PBS pH 7.4 + 0.5% Triton X-100 (2 × 10 min) and incubated in the same solution containing 2% BSA and 10% normal goat serum for 1 h at RT (blocking solution). The tissues were then incubated with primary antibodies (anti-HuC/D and anti-VIP for submucosal layer whole-mount preparations and anti-VIP for myenteric layer whole-mount preparations) in PBS + 0.5% Triton X-100 + 2% BSA + 2% normal goat serum for 2 h in an incubator at 37°C, and then incubated at RT for 46 h with slow agitation. Next, three washes (5 min) in PBS + 0.5% Triton X-100 were performed and the tissues were incubated for 1 h at RT with the secondary antibody (Table 1). The whole-mount preparations were then washed with 0.1 M PBS, pH 7.4 (3 × 5 min), slide-mounted with Prolong Gold antifade, and stored at 4°C. These whole-mount immunostains were used to assess the neuronal population (HuC/D) in the two plexuses, subpopulation of submucosal VIP-containing neurons and myenteric VIP-containing nerve fiber varicosities.

#### Calcitonin gene-related peptide (CGRP)-immunoreactive nerve fibers

CGRP, a marker of intrinsic primary afferent neurons (IPANs), was detected using immunostaining to identify CGRP-IR nerve fiber varicosities within myenteric and submucosal plexuses. These nerve fibers originate from intrinsic and extrinsic sensory neurons [23]. Myenteric and submucosal layer whole-mount preparations were subjected to a series of washes. After blocking (similar protocol to HuC/D and VIP double staining), the tissues were separately incubated with the primary antibody as shown in Table 1 (anti-CGRP). After washing with 0.1 M PBS pH 7.4 (3 × 5 min), the tissues were incubated with the secondary antibody (Table 1) at RT for 1 h and washed in 0.1 M PBS pH 7.4 (3 × 5 min). The whole-mount preparations were then slide-mounted with Prolong Gold antifade and stored at 4°C.

### Quantitative Analysis and Neuronal Density

Quantitative analysis was performed by random sampling at the intermediary region of the jejunum and ileum circumferences (60–120° and 240–300°, considering 0° as the mesenteric intersection). Thirty images per animal from each intestinal segment were acquired with a 20× objective using an Axiocam high-resolution camera (Zeiss, Oberkochen, Germany) coupled to an Axioskop Plus light microscope with immunofluorescence filters (Zeiss, Oberkochen, Germany) and digitized with a microcomputer and the Axiovision version 4.1 software [31, 35]. For quantitative analysis of the myenteric and submucosal plexuses, all neurons in each image were counted. Quantification and determination of the image area were performed using the Image Pro^®^ Plus 4.5 image analysis software (Media Cybernetics, Inc., Silver Spring, MD, USA). Neuronal density for the general population and for each neuronal subpopulation was expressed as total neurons per square centimeter.

### Morphometric Analysis of Neurons and Varicosities

For the morphometric analysis of neuronal cell bodies, somatic areas (in μm^2^) of 100 neurons from each animal were measured for all immunohistochemistry techniques. For the morphometric evaluation of varicosities (small and frequent neurotransmitter-containing dilations along the fibers), areas (in μm^2^) of 250 varicosities per animal were measured from images acquired with a 40× objective. Image Pro^®^ Plus 4.5 image analysis software was used for the morphometric analysis of all images.

### Western Blot Quantitative Analysis

Expression of ChAT, VIP, and CGRP proteins was quantified in the jejunum and ileum by Western blotting. Following laparotomy, the harvested segments were repeatedly washed with Krebs-Ringer buffer solution (pH 7.4) to completely remove the feces. The tissue was lysed by immersion in homogenization buffer (50 mM Tris HCl pH 7.2, 600 mM NaCl, 1 mM ethylenediaminetetracetic acid (EDTA) Sigma-Aldrich, Inc.) containing protease inhibitor. Lysed samples were then centrifuged for 10 min at 10,000 × *g* to remove the insoluble fraction. The supernatant was collected and frozen (−80°C) until further use. Supernatant total protein concentration was determined using the Bradford method (Bio-Rad, Hercules, CA, USA) [37]. In parallel, an equivalent fraction from each sample (30 μg) was separated by 15% SDS-PAGE (VIP and CGRP) or 12% SDS-PAGE (ChAT). The electrophoresis bands were then transferred to nitrocellulose membranes (Bio-Rad). After blocking with 5% skim milk in TBS buffer (2.24 g/L Tris base and 8 g/L NaCl, pH 7.6) with 0.1% Tween-20 for 1 h at RT, the membranes were incubated (overnight at 4°C) with primary antibodies against ChAT, VIP, and CGRP proteins (Table 1). The membranes were then incubated for 2 h at RT with a secondary peroxidase-conjugated antibody (Table 1). Secondary antibody detection employed a Novex ECL Chemiluminescent Substrate Reagent Kit (Thermo Fisher Scientific, Waltham, MA, USA), a detection system for Western blotting. Membrane chemiluminescence was detected using the ChemiDoc MP imaging system, which captures images for posterior analysis, according to the manufacturer’s instructions (V3 Western Workflow^™^, Bio-Rad). The bands obtained from the images corresponded to the ChAT (70 kDa), VIP (17 kDa), and CGRP (15 kDa) proteins and were quantified using ImageJ software (National Institutes of Health, Bethesda, MD, USA). Changes in the expression of proteins analyzed by Western blotting in this study were normalized to the expression of glyceraldehyde-3-phosphate dehydrogenase (GAPDH). All protocols for electrophoresis and Western blotting were performed according to the Bio-Rad Mini Protean System standard procedures.

### Correction of Neuron Density

In order to prevent morphoanatomical changes, which occur in several physiopathological conditions and interfere with the neuronal density results (neuron dispersion or concentration), a correction factor was applied for each intestinal segment (jejunum and ileum) in each experimental group [38, 39]. By comparing mean areas with the control group, a correction factor was calculated as the ratio between the areas of the intestinal segments in the experimental groups (CG, TW, and TWG) and in the control group (Table 2) and was applied to correct the results of the quantitative analysis [39, 40]. Therefore, only the corrected neuronal density data are presented.

**Table 2:** Average area of the small intestine and correction factors applied for the of neuronal density correction in the myenteric and submucosal plexuses of the jejunum and ileum. Experimental groups: control (C); control supplemented with 2% L-glutamine (CG); Walker-256 tumor (TW); and Walker-256 tumor supplemented with 2% L-glutamine (TWG). n = 8 rats per group.

	Jejunum area (cm^2^)	Correction factors	Ileum area (cm^2^)	Correction factors
**C**	113.7 ± 3.5	Standard	113.6 ± 2.9	Standard
**CG**	110.2 ± 6.0	0.969	110.64 ± 5.1	0.974
**TW**	76.7 ± 3.9*	0.675	90.7 ± 2.6*	0.799
**TWG**	80.5 ± 4.6*	0.701	100.8 ± 2.2*	0.887

Data are expressed as mean ± SEM.

* represents significant difference (p < 0.001) compared with control group according to ANOVA (two way) followed by Tukey's post hoc test.

### Statistical Analysis

Statistical analysis was performed using Statistica 7.0 (StatSoft) and GraphPad Prism 6.0 software. (GraphPad Software)The data were presented as the mean ± standard error of the mean (SEM). Analysis of variance (two-way ANOVA) was employed for all data. The Tukey’s *post-hoc* test was applied whenever ANOVA indicated a significant difference. The unpaired Student’s *t*-test was employed for comparisons between two groups. The significance level was defined as p < 0.05.

## Results

### Physiological Parameters

Subcutaneous implantation of Walker 256 tumor cells promoted cachexia due to growth of a solid tumor in the right flank in the TW group (Table 3). Significant evidence of cachexia included a total body weight decrease of 9% in the TW group compared to in the control group (p = 0.0288; Table 3). Dietary l-glutamine (2%) supplementation (TWG group) had a positive effect on this parameter, preventing the decrease in the average total body weight (TWG vs. TW; p = 0.0399; Table 3) and maintaining these values close to those measured in groups C and CG (p = 0.1927; Table 3). The tumor mass was substantial in animals from the TW and TWG groups, accounting for 13% and 10%, respectively, of total body weight in these groups. l-glutamine did not favor tumor growth, but rather decreased tumor growth by approximately 16%, although the difference was not statistically significant (TW vs. TWG; p = 0.3946; Table 3). Based on the cachexia evaluation criteria of weight variation (WV), the TW and TWG groups showed positive variation in this parameter. However, compared to each other, the l-glutamine-supplemented group showed a 54% greater weight variation compared to the non-supplemented tumor-bearing group (TWG vs. TW; p = 0.0106; Table 3). The results for the cachexia index calculated for both groups (TW and TWG) revealed that dietary supplementation with l-glutamine significantly decreased this index by 5.1% (p = 0.0479; Table 3). There was no statistically significant difference in food intake (g/100 g body mass/day) among the groups (p = 0.4468; Table 3).

**Table 3:** Physiological parameters assessed in experimental groups. Final weight body (FW), food intake (FI), tumor mass (TM), weight variation (WV). Experimental groups: control (C), control supplemented with L-glutamine (CG), Walker 256 tumor (TW) and Walker 256 tumor supplemented with L-glutamine (TWG). n = 8 rats per group.

	FW (g)	FI	TM (g)	CI (%)	WV(g)
**C**	287.0 ± 5.5	8.1± 0.2			
**CG**	285.7 ± 6.9	8.1 ± 0.3			
**TW**	259.9 ± 5.2*	8.4 ± 0.3	33.4 ± 3.8	17.8 ± 1.6	+30.1 ± 3.8
**TWG**	276.1 ± 4.0**	7.7 ± 0.3	28.0 ± 4.9	12.6 ± 1.8^#^	+46.4± 4.0^#^

Values expressed as mean expressed as mean ± SEM

CI (%) = (initial body mass—final body mass + tumor mass + weight body mass gain of control)/(initial body mass + body mass gain of control)] × 100% [5]

FI = g/100g weight body/day

^+^ represents positive weight variation

* Indicates a significant difference when compared with C group (p < 0.05) by analysis of variance (two-way ANOVA) followed by Tukey's post hoc test

** Indicates a significant difference from TW group (p < 0.05) determined by ANOVA (two-way) followed by Tukey's post hoc test

^#^ Indicates a significant difference from TW group (p < 0.05) according student t test

### Neuronal Quantitative Analysis

A significant decrease in the small intestine area was observed in the experimental model animals with cancer cachexia (TW and TWG groups). We took this change in area into account when assessing changes in neuron numbers. The correction of neuronal density is required to minimize errors and biases, including normalization by the size of the tissue or organ [40]. After the correction factor was applied, neuronal density was expressed as neuron number per square (Tables 4 and 5). The neuronal density (uncorrected data) of myenteric and submucosal plexuses in the jejunum and ileum can be found in S1 and S2 Tables.

**Table 4:** Neuronal density of myenteric neurons (neurons/cm^2^) in the jejunum and ileum. Experimental groups: control (C); control supplemented with 2% L-glutamine (CG); Walker-256 tumor (TW); and Walker-256 tumor supplemented with 2% L-glutamine (TWG). HuC/D-IR population (neurons/cm^2^), CHAT-immunoreactive subpopulation (neurons/cm^2^).

		Experimental Groups			
		C	CG	TW	TWG
**Jejunum**	HuC/D	14986.3 ± 499.9^a^	16216.5 ± 393.5^a^	10379.9 ± 319.4^b^	10822.9 ± 276.6^b^
	CHAT	11233.9 ± 483.4^a^	12130.7 ± 225.3^a^	7050.2 ± 212.7^b^	7807.7 ± 350.4^b^
**Ileum**	HuC/D	20843.9 ± 835.4^a^	21353.0 ± 459.7^a^	17225.4 ± 676.6^b^	20839.9 ± 472.5^a^
	CHAT	15270.9 ± 606.1^a^	15900.5 ± 517.8^a^	13151.5 ± 415.4^b^	15213.8 ± 574.8^a^

The results are expressed as mean ± SEM (n = 8).

Different letters in the same row indicate significant difference (p<0.05) according to ANOVA (two-way) followed by Tukey's *post hoc* test.

**Table 5:** Neuronal density of submucous neurons (neurons/cm^2^) in the jejunum and ileum. Experimental groups: control (C); control supplemented with 2% L-glutamine (CG); Walker-256 tumor (TW); and Walker-256 tumor supplemented with 2% L-glutamine (TWG). HuC/D-IR population (neurons/cm^2^) and VIP-immunoreactive subpopulation (neurons/cm^2^) densities.

	Experimental Groups				
		C	CG	TW	TWG
**Jejunum**	HuC/D	9451.1 ± 154.3^a^	9900.6 ± 249.7^a^	6546.2 ± 158.1^b^	6599.8 ± 118.5^b^
	VIP	4234.9 ± 121.1^a^	4567.9 ± 86.3^a^	3512.4 ± 79.8^b^	3411.3 ± 97.2^b^
**Ileum**	HuC/D	8738.6 ± 268.5^a^	8744.1 ± 216.6^a^	7836.5 ± 176.0^b^	6954.2 ± 183.7^b^
	VIP	3985.6 ± 78.2^a^	4018.4 ± 88.4^a^	3429.9 ± 97.0^b^	3246.2 ± 93.6^b^

The results are expressed as mean ± SEM (n = 8).

Different letters indicate significant difference (p<0.05) according to ANOVA (two-way) followed Tukey's post hoc test.

### Neuronal Density in the Myenteric Plexus: HuC/D-IR Neurons

**Fig 1:**
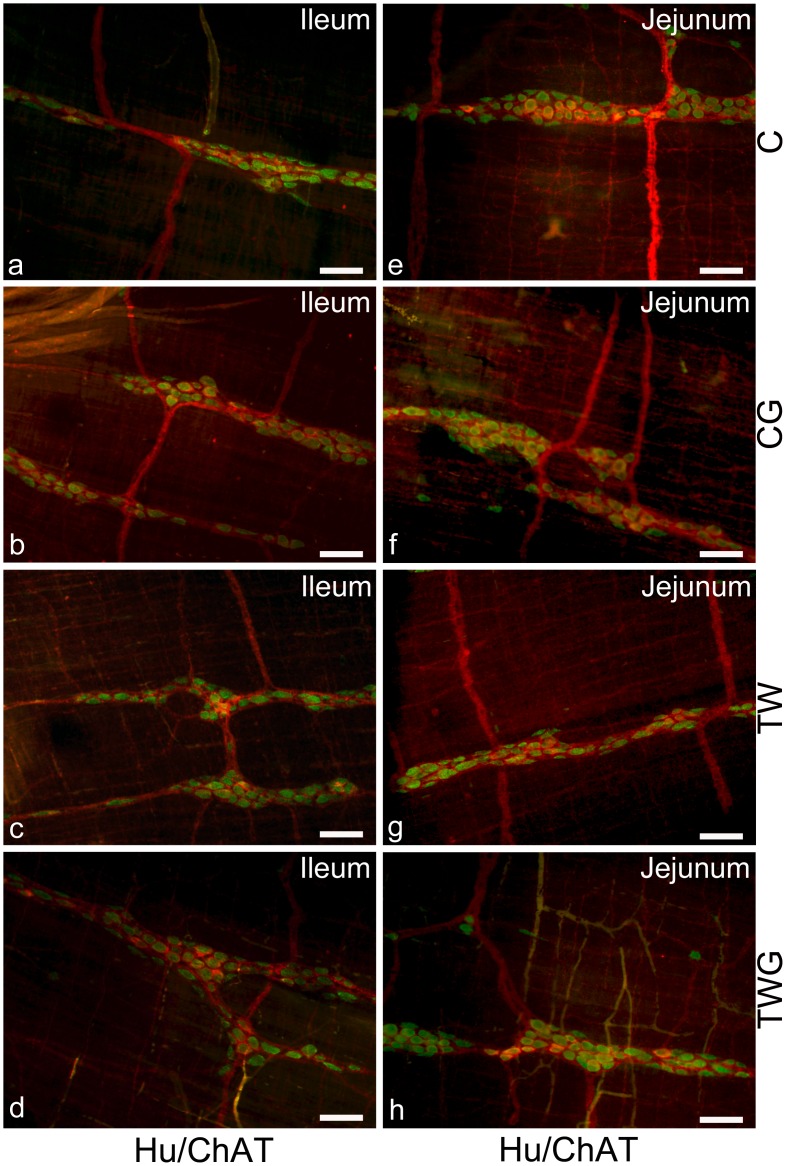
Representative images of Hu/CHAT myenteric neurons. Double immunostaining of HuC/D and CHAT myenteric neurons of ileum (a-d) and jejunum (e-h). Experimental groups: control (C); control supplemented with 2% L-glutamine (CG); Walker-256 tumor (TW); and Walker-256 tumor supplemented with 2% L-glutamine (TWG). These images are composites of merged images taken separately from red (CHAT) and green (HuC/D) fluorescent channels. Scale Bar 50 μm.

All experimental groups were compared with each other using double immunostaining preparations for HuC/D-IR and CHAT-IR neurons in the ileum (Fig 1a–1d) and jejunum (Fig 1e–1h). The myenteric plexus data indicated a significant decrease in the density of HuC/D-IR neurons in both the jejunum (30.7%, p = 0.0002, Table 4) and ileum (17.3%, p = 0.0017, Table 4) 14 days after inoculation of Walker 256 carcinosarcoma cells (TW vs. C; Table 4). Supplementation with l-glutamine (TWG group) did not prevent the decrease in neuronal density in the jejunum (TWG vs. TW; p = 0.7905; Table 4). However, in the ileum, l-glutamine supplementation maintained neuronal density values very close to those observed in the C and CG groups (p = 1.000 and p = 0.9207; Table 4). No change was detected in the CG group compared with the C group in neuronal density for HuC/D-IR (p = 0.0775 and p = 0.9282; Table 4)

### Neuronal Density in the Myenteric Plexus: ChAT-IR Neurons

Quantitative analysis of the cholinergic subpopulation in the myenteric plexus showed that the presence of cancer caused a marked decrease in the number of ChAT-immunoreactive cell bodies compared to control in the jejunum (p = 0.0002; S2 Fig) and ileum (p = 0.0277; S1 Fig), with values of 37.2% and 13.8%, respectively (Table 4). In the TWG group, a higher percentage of ChAT-IR neurons per area was observed in the jejunum (11%) compared to the TW group. However, this difference was not significant (p = 0.2930, Table 4). In the ileum, in contrast l-glutamine prevented the decrease in ChAT-IR neuronal density (TWG vs. TW; p = 0.0332, Table 4), with the density remaining similar to that observed in the C and CG groups (p = 0.9998 and p = 0.7538, Table 4). No change was detected in the CG group compared with the C group for CHAT-IR neuronal density in the jejunum (p = 0.1700, Table 4) and ileum (p = 0.7993, Table 4). The ratio of ChAT-IR neurons to HuC/D-IR myenteric neurons was 75% and 74% in the jejunum for C and CG groups, respectively, and decreased to approximately 68% in TW group. During supplementation (TWG), l-glutamine prevented this reduction in ratio, which was 72% of the total neuronal population. In the ileum, the proportion of ChAT-IR myenteric neurons was similar among all the groups, approximately 73% (C), 72.5% (CG), 76% (TW), and 73% (TWG).

### Neuronal Density in the Submucosal Plexus: HuC/D-IR Neurons

**Fig 2:**
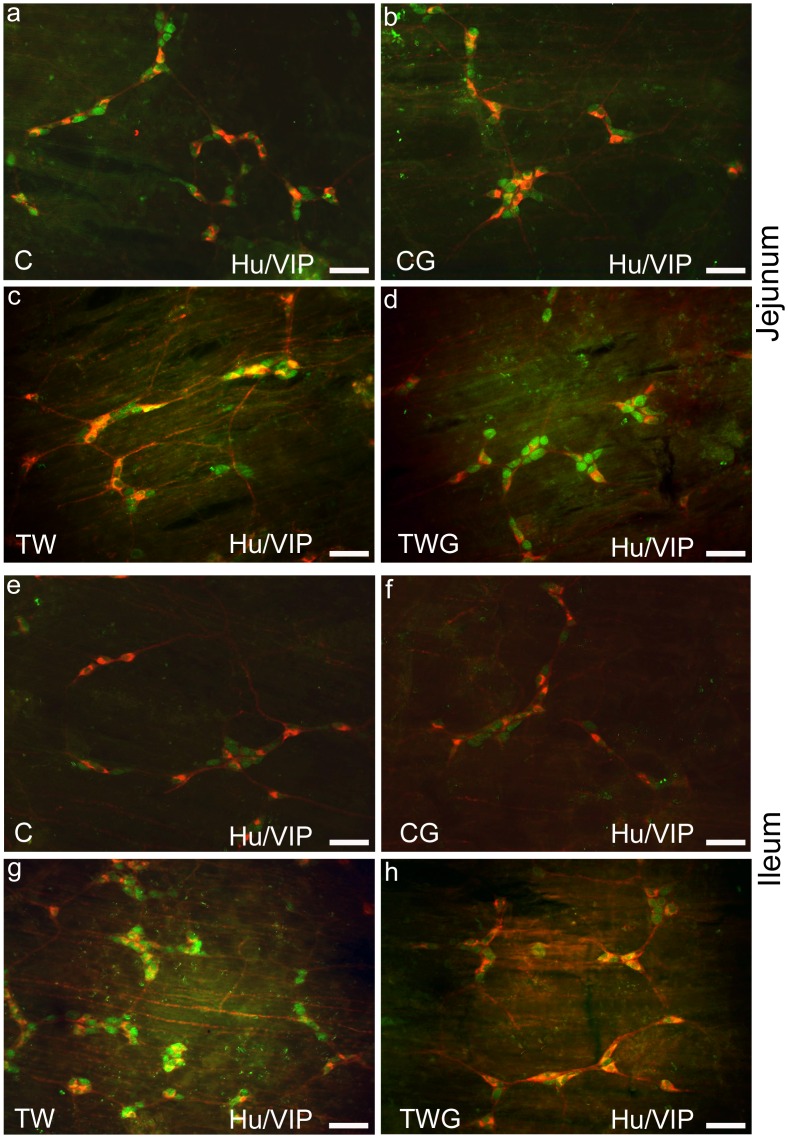
Representative photomicrographs of Hu/VIP submucous neurons. Double immunostaining of HuC/D and VIP submucous neurons of jejunum (a-d) and ileum (e-f) from experimental groups: control (C); control supplemented with 2% L-glutamine (CG); Walker-256 tumor (TW); and Walker-256 tumor supplemented with 2% L-glutamine (TWG). These images are composites of merged images taken separately from red (VIP) and green (HuC/D) fluorescent channels. Scale Bar 50 μm.

Double immunostaining for HuC/D and VIP were used for submucous neuronal localization and morphoquantitative analysis in the jejunum (Fig 2a–2d) and ileum (Fig 2e–2h) in all the experimental groups. HuC/D-IR neuronal density was significantly reduced in the presence of cancer (TW group) by 30.3% in the jejunum (p = 0.0002) and 9.9% in the ileum (p = 0.0486) compared to the control group (Table 5). In the TWG group, the neuronal density decrease was not prevented by l-glutamine supplementation in either of the intestinal segments examined (TWG vs. TW; jejunum; p = 0.9958 and ileum; p = 0.0552, Table 5). Specifically, in the ileum, the TWG group displayed an 11.2% decrease in neuronal density compared to the TW group (Table 5).

### Neuronal Density in the Submucosal Plexus: VIP-IR Neurons

The VIPergic subpopulation in the submucosal plexus was 17.1% (p = 0.0002, Table 5) and 14.0% (p = 0.0023, Table 5) lower in the TW group than in the C group in the jejunum and ileum, respectively. Very similar VIP-IR neuron proportions were observed for the jejunum (44.8%) and ileum (45.6%) in control rats. Submucosal VIP-IR neurons in the TW group, only in the jejunum, proportionally increased to approximately 54%, while in the ileum the proportion was 44%. In the TWG group, for both intestinal segments, l-glutamine did not significantly prevent the decrease in VIP-IR neuronal density when compared to the TW group (jejunum; p = 0.8196 and ileum; p = 0.5259, Table 5). In this plexus, the ratio of VIP-IR neurons to HuC/D neurons in TWG rats was 52% and 47%, respectively, for the jejunum and ileum.

### Neuronal Cell Body Morphometric Analysis: HuC/D-IR and ChAT-IR Myenteric Neurons

For both assessed segments, the average cell body area of HuC/D-IR neurons decreased in the myenteric plexus because of the presence of the Walker 256 tumor (jejunum and ileum; TW vs. C; p < 0.0001, Table 6). The neuronal body area decreased by approximately 15% and 13% in the jejunum and ileum, respectively. l-glutamine (TWG) prevented the cell body area reduction of HuC/D-IR neurons in the jejunum and ileum (TWG vs. TW; p < 0.0001, Table 6). The cell body area of ChAT-IR neurons in the jejunum decreased in TW rats compared to that in control rats (TW vs. C; p < 0.0001, Table 6). After supplementation, ChAT-IR neurons in the TWG group displayed a 28.5% larger average cell body area compared to that in the TW group (p < 0.0001, Table 6). Their area was also larger than that observed in the C and CG groups (p < 0.0001, Table 6). In the ileum, no difference was detected among the groups (p > 0.05, Table 6) in the average cell body area of cholinergic neurons. In addition, the C and CG groups showed no difference in morphometric analysis of both HuC/D-IR (jejunum; p = 0.0698 and ileum; p = 0.7475, Table 6) and ChAT-IR neurons in the two segments (jejunum; p = 0.8695 and ileum; p = 0.9494, Table 6).

**Table 6:** Neuronal morphometric analysis of myenteric plexus. Neuronal cell body area (μm^2^) of Jejunum and ileum from groups: control (C); control supplemented with 2% L-glutamine (CG); Walker-256 tumor (TW); and Walker-256 tumor supplemented with 2% L-glutamine (TWG).

	Experimental Groups				
		C	CG	TW	TWG
**Jejunum**	HuC/D	327.3 ± 4.4^a^	340. 9 ± 4.5^a^	283.8 ± 4.2^b^	304.6 ± 4.7^c^
	CHAT	284.5 ± 4.4^a^	280.1 ± 4.5^a^	254.5 ± 4.7^b^	327.1 ± 4.2^c^
**Ileum**	HuC/D	305.1 ± 4.0^a^	298.9 ± 4.7^a^	269.6 ± 3.8^b^	298.8 ± 4.8^a^
	CHAT	293.5 ± 4.2^a^	290.2 ± 3.9^a^	307.7 ± 4.2^a^	294.3 ± 4.5^a^

All the values are expressed as mean ± SEM. n = 8 animals per group.

Significant differences are represented by different letters in same row (p < 0.001) have been obtained with the two-way ANOVA followed by Tukey's post hoc test.

### Neuronal Cell Body Morphometric Analysis: HuC/D-IR and VIP-IR Submucous Neurons

Surprisingly, in the submucosal plexus, cachexia caused an increase in the cell body area of HuC/D-IR neurons in the jejunum (TW vs. C; p < 0.0001, Table 7), while, in the ileum, no significant difference was observed among the groups (p > 0.05, Table 7). In the jejunum of the TWG group, the neuron cell body area (HuC/D) was larger than that of the TW group, with l-glutamine supplementation displaying no preventative effect (TWG vs. TW; p < 0.0001, Table 7).

**Table 7:** Neuronal morphometric analysis of submucosal plexus. Neuronal cell body area (μm^2^) of jejunum and ileum from groups: control (C); control supplemented with 2% L-glutamine (CG); Walker-256 tumor (TW); and Walker-256 tumor supplemented with 2% L-glutamine (TWG).

		Experimental Groups			
		C	CG	TW	TWG
**Jejunum**	HuC/D	284.6 ± 3.4^a^	274.7 ± 3.7^a^	299.2 ± 3.6^b^	314.7 ± 3.2^c^
	VIP	291.6 ± 3.3^a^	279.9 ± 2.9^a^	336.1 ± 4.6^b^	284.5 ± 3.2^a^
**Ileum**	HuC/D	276.8 ± 4.0^a^	273.3 ± 3.7^a^	265.9 ± 3.7^a^	267.5 ± 3.5^a^
	VIP	301.2 ± 4.8^a,c^	289.1 ± 3.7^a,^	328.7 ± 4.4^b^	305.8 ± 3.6^c^

All the results are expressed as mean ± SEM (n = 8 animals per group).

Means followed by different letters in the same row are significantly different according to ANOVA (two-way) followed Tukey's post hoc test (p < 0.001).

The average area of the VIP-IR neuron subpopulation in the submucosal plexus of the TW group was significantly larger than that of the C group for both intestinal segments (p < 0.0001, Table 7). In the TWG group, l-glutamine prevented the increase in neuronal cell body area in this subpopulation (TWG vs. TW; p < 0.0001, Table 7) and maintained neuron size both in the jejunum (TWG vs. C; p = 0.4984, Table 7) and in the ileum (TWG vs. C; p = 0.8576, Table 7), similar to those of the control group. No difference was observed for the C and CG groups (jejunum; p = 0.0951 and ileum p = 0.1695, Table 7).

### Morphometric Analysis of Myenteric CGRP-IR and VIP-IR Varicosities

**Fig 3:**
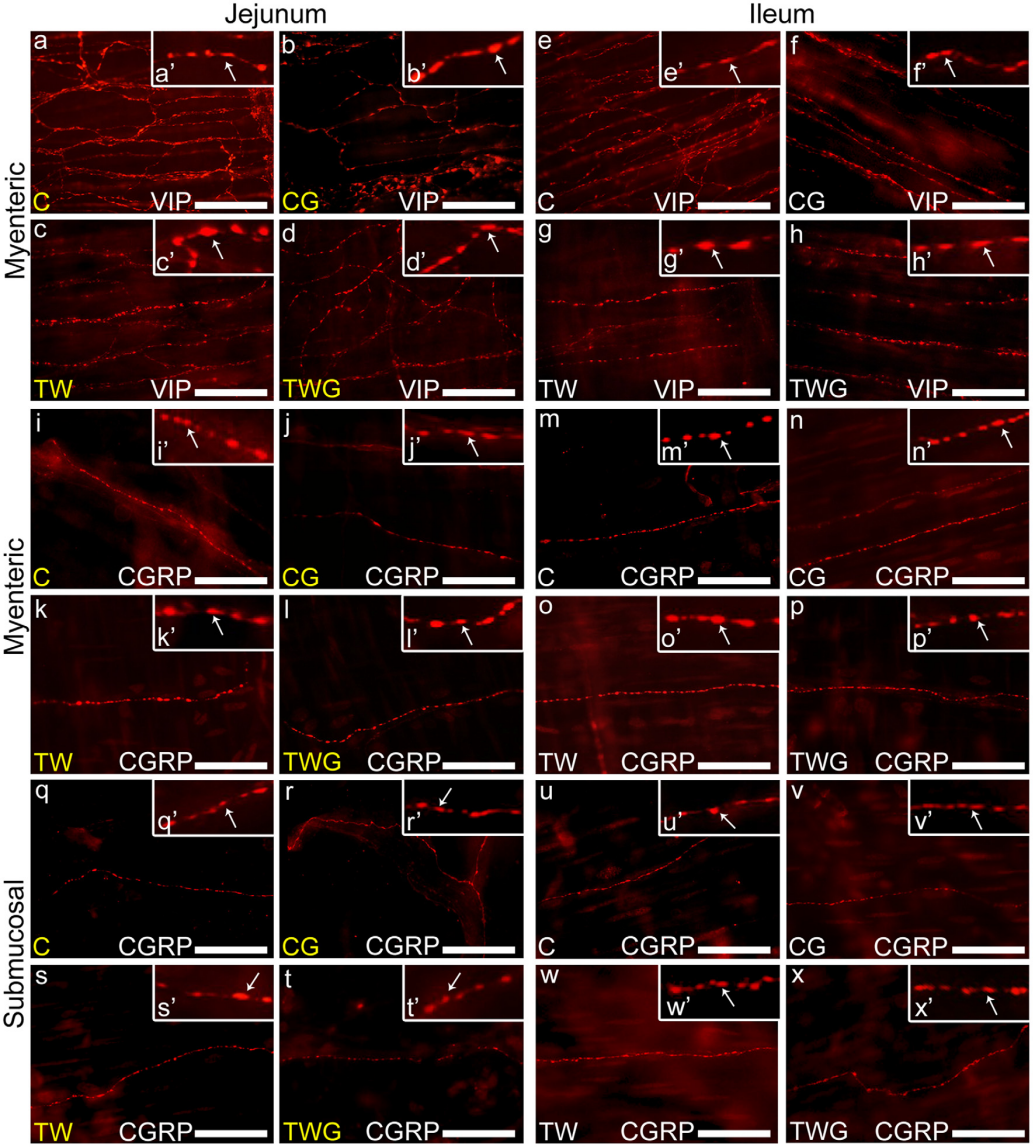
Representative images of the myenteric varicosities nerve fibers. Myenteric VIP-IR and CGRP-IR varicosities of the jejunum: (a-d) VIP-IR nerve fiber and (i-l) CGRP-IR nerve fiber. Ileum: (e-h) VIP-IR nerve fiber and (m-p) CGRP-IR nerve fiber. Experimental groups: control (C); control supplemented with 2% L-glutamine (CG); Walker-256 tumor (TW); and Walker-256 tumor supplemented with 2% L-glutamine (TWG). (q-x) representative images of the submucous CGRP-IR varicosities nerve fibers of the jejunum (q-t) and ileum (u-x) from experimental groups (C, CG, TW and TWG). All enlarge images on the top right of each image with white arrows indicate examples of immunoreactive varicosity (a'-x'). Scale Bar 25 μm.

In both the jejunum and ileum of TW rats, the areas of VIP-IR (Fig 3a–3h) and CGRP-IR (Fig 3i–3p) myenteric nerve fibers varicosities increased (TW vs. C; p < 0.0001, Table 8). l-glutamine supplementation efficiently prevented the increase in the area of fiber varicosities (VIP-IR and CGRP-IR) in both segments (TWG vs. TW; p < 0.0001, Table 8). Compared to the C and CG groups, there was no significant difference in the areas of VIP-IR (C vs. CG; p = 0.1262, Table 8) and CGRP-IR (C vs. CG; p = 0.2328, Table 8) varicosities in the jejunum. However, in the ileum, the difference between the two groups was significant (C vs. CG; p < 0.0001, Table 8), highlighting the effect of l-glutamine in increasing the areas in the varicosities in the CG group.

**Table 8:** Morphometric analysis of VIP-immunoreactive and CGRP-immunoreactive varicosities areas (μm^2^) in the myenteric plexus in the intestinal regions (jejunum and Ileum). Experimental groups: control (C); control supplemented with 2% L-glutamine (CG); Walker-256 tumor (TW); and Walker-256 tumor supplemented with 2% L-glutamine (TWG).

	Experimental Groups				
		C	CG	TW	TWG
**Jejunum**	CGRP	2.8 ± 0.1^a^	2.9 ± 0.1^a^	3.1 ± 0.1^b^	2.9 ± 0.1^a^
	VIP	2.6 ± 0.1^a^	2.7 ± 0.1^a^	4.3 ± 0.1^b^	3.3 ± 0.1^c^
**Ileum**	CGRP	1.9 ± 0.1^a^	2.1 ± 0.1^b^	2.6 ± 0.1^c^	2.2 ± 0.1^b^
	VIP	2.8 ± 0.1^a^	3.2 ± 0.1^b^	3.7 ± 0.1^c^	3.3 ± 0.1^b^

All the values are expressed as mean ± SEM (n = 8 animals per group).

Different letters in the same row indicate significantly different (p < 0.001) according to ANOVA (two-way) followed Tukey's post hoc test.

### Morphometric Analysis of Submucosal CGRP-IR Varicosities

In the submucosal plexus, the areas of CGRP-immunoreactive varicosities increased in the TW group (TW vs. C, p < 0.0001, Table 9) in both the jejunum (Fig 3q–3t) and ileum (Fig 3u–3x). Dietary l-glutamine supplementation to tumor-bearing rats (TWG) was effective in preventing an increase in the size of the varicosities of this plexus in the jejunum (TWG vs. TW, p = 0.0002, Table 9) and ileum (TWG vs. TW, p < 0.0001, Table 9). In the jejunum, the area of CGRP-IR varicosities of the CG group increased compared to that in the control group (p = 0.0002 Table 9). However, in the ileum, there was no significant difference in the areas (C vs. CG, p = 0.7410, Table 9).

**Table 9:** Morphometric analysis of CGRP-immunoreactive varicosities areas (μm^2^) in the submucosal plexus in the jejunum and Ileum from the following groups: control (C); control supplemented with 2% L-glutamine (CG); Walker-256 tumor (TW); and Walker-256 tumor supplemented with 2% L-glutamine (TWG).

	Experimental Groups				
		C	CG	TW	TWG
**Jejunum**	CGRP	1.7 ± 0.1^a^	1.8 ± 0.1^b^	2.1 ± 0.1^c^	1.9 ± 0.1^b^
**Ileum**	CGRP	1.4 ± 0.1^a^	1.4 ± 0.1^a^	1.6 ± 0.1^b^	1.5 ± 0.1^c^

All the values are expressed as mean ± SEM (n = 8 animals per group).

Different letters in the same row are significantly different (p < 0.001) according to ANOVA (two-way) followed Tukey's post hoc test.

### Western Blot Analysis of ChAT Enzyme and VIP and CGRP Neuropeptide Expression

**Fig 4:**
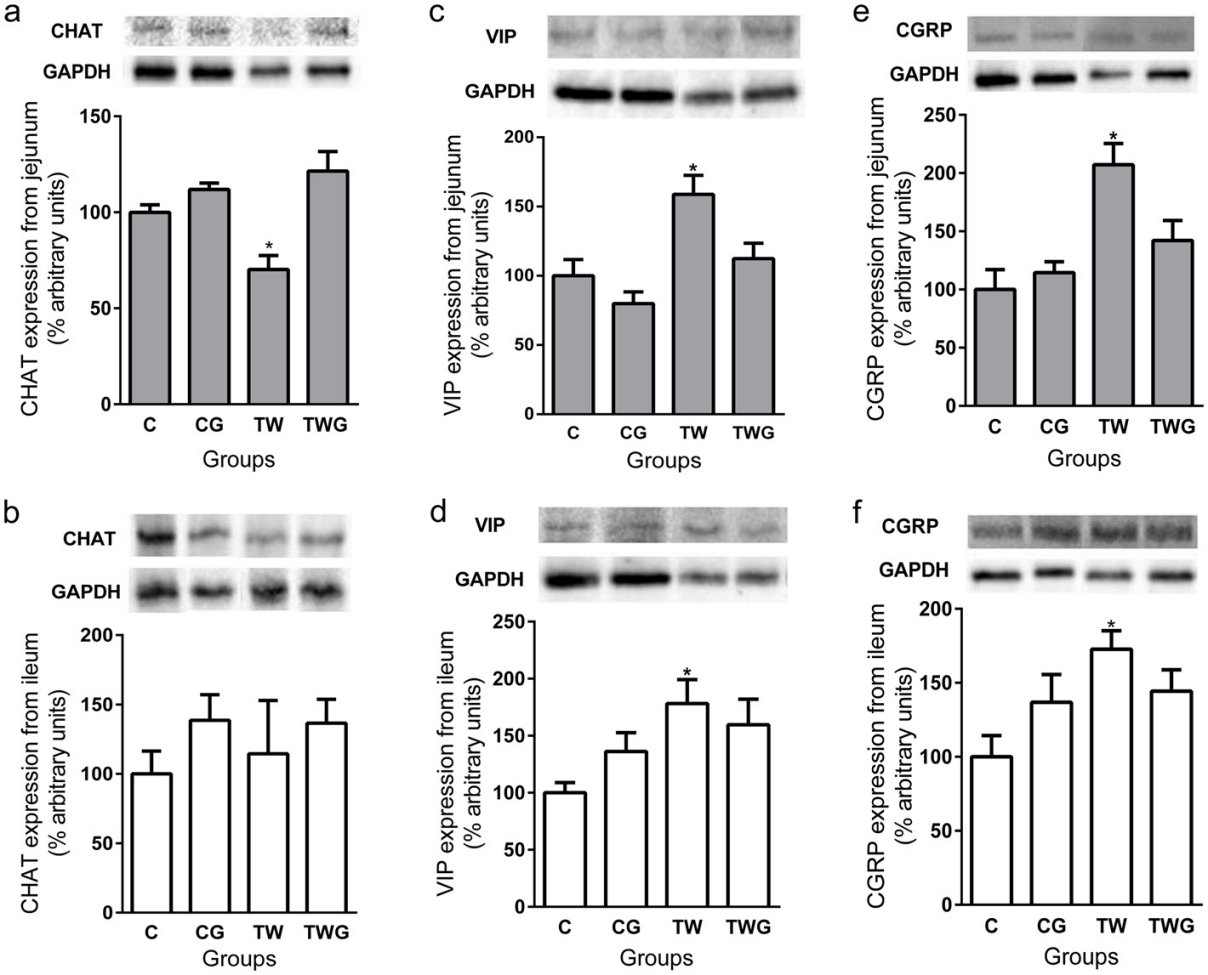
Effect of supplementation of L-glutamine and cachexia on proteins (CHAT, VIP and CGRP) expression from jejunal and ileal samples of each experimental group. Groups: control (C); control supplemented with 2% L-glutamine (CG); Walker-256 tumor (TW); and Walker-256 tumor supplemented with 2% L-glutamine (TWG). (a) Jejunum CHAT expression and representative bands (on the top). (b) Ileum CHAT expression and representative bands (on the top). (c) Jejunum VIP expression and representative bands (on the top). (d) Ileum VIP expression and representative bands (on the top). (e) Jejunum CGRP expression and representative bands (on the top). (f) Ileum CGRP expression and representative bands (on the top). Bars represent means ± SEM of samples from four animals. The data are presented as percentage arbitrary units (% of control) after normalization (GAPDH). * indicates significant difference (*p* < 0.05) versus control group.

The results indicated a decreased expression of the ChAT enzyme in the TW group in the jejunum (29.8%) compared to that in the C group (p = 0.0390, Fig 4a). In the TWG group, l-glutamine prevented the decreased of the ChAT enzyme (TWG vs. TW; p = 0.0002, Fig 4a).

In the ileum samples, no significant difference was detected among groups (CG, TW, TWG vs. C; p > 0.05, Fig 4b). Neuropeptide VIP levels in TW group samples from both the jejunum (p = 0.0390, Fig 4c) and ileum (p = 0.0410, Fig 4c) were significantly higher than that in the control group. l-glutamine decreased VIP expression to levels similar to those in the control group in the jejunum (p = 0.1667 Fig 4c), while in the ileum, a 25% decrease in VIP expression with supplementation was observed. However, the difference was not statistically significant (p = 0.1412, Fig 4d). Significantly increased levels of CGRP were observed in the TW group for the jejunum (p = 0.0023, Fig 4e) and ileum samples (p = 0.0245, Fig 4f). However, in the supplemented group (TWG), these levels remained similar to those in the control group (jejunum; p = 0.2879 and ileum; p = 0.2202, Fig 4e and 4f) for both intestinal segments.

## Discussion

### Cachexia Parameters

After a 14-day experimental period, a cachexia condition in Walker 256 tumor-bearing rats was confirmed by changes in pathophysiological parameters. Cachexia is a typical occurrence in this experimental model, and our results are consistent with those of other studies using this model [5, 34]. As cancer develops, the demand for several nutrients increases; this is particularly seen for l-glutamine, which is highly metabolized by cancer cells during rapid cell proliferation [11, 41]. Supplying TW rats with l-glutamine may have influenced tumor growth and worsened the cachectic condition, as several cancer cell types depend on this amino acid [10, 11]. However, the supplementation had no effect on tumor mass and showed consistent results with previous reports regarding l-glutamine use on cachexia, muscle damage and weight loss prevention [10, 30, 41].

### Cachexia, Small Intestine Size, and Correction of Neuronal Density

Neoplastic cachexia caused a decrease in the total area of the intestinal segments, that is, 33% for the jejunum and 20% for the ileum. Consistent with this, two studies reported a 18% and 15% decrease, respectively, in the length of the small intestine and a 26% decrease in the villus height in Walker tumor model, indicating the consequences of cachexia [11, 42]. This decrease may be an adaptation to the pathogenic factors of cachexia, functionally altering the organ and its neural components.

Changes in total area of the small intestine may reflect apparent estimations of the neuronal density and to minimize this effect a correction factor proportional to the segment intestine shortening was applied [40]. This effect can be handled by determining an estimate total number of neurons within an organ, by measures such as neurons per ganglion or per ganglionic area, and by determining a size correction factor for any gut regions [40]. Whereas the first two approaches are quite problematic [40], we adjusted neuronal density for each intestinal segment as previously described [39, 40, 43, 44]. The behavior of corrected neuronal density indicate a decrease in neurons number in cancer cachexia.

### Alterations in Enteric Neurons from Cancer-Related Cachexia

Walker 256 tumor has been used as cachexia experimental model and has shown all features that are commonly seen in cachectic patients [34], including both the presence of inflammatory mediators [45] and oxidative stress markers [27]. In this model some studies have pointed to systemic [28] and tissue localized oxidative stress [5], including in several neural tissues in the central nervous system (CNS) [27, 46]. Taken together this evidence suggests that extra-tumoral tissues may also be affected by tumor-derived factors [16], which may damage neural components within the intestine. The mechanism behind the neuronal damage is probably related to mediated oxidative stress, since it plays a pivotal role in neuronal injury in the ENS [6]. Several previous studies in different experimental models of pathological conditions [31, 33, 38, 47], have associated the decrease of neuronal density in the ENS with oxidative damage. Therefore, in the current study the decrease in neuronal density of the pan neuronal population (HuC/D-IR) and ChAT-IR and VIP-IR subpopulations may result from systemic effects of cachexia, mediated by oxidative stress. Therefore, oxidative stress methods are suitable for investigating this condition within the ENS in this experimental model.

In the myenteric plexus of the rat small intestine, cholinergic neurons constitute the major subpopulation, comprising 75% of the total [15] according to the present study and others [47, 48]. The proportional decrease of ChAT-containing neurons in this plexus was followed by somatic atrophy and a decrease in ChAT expression due to cachexia. These changes may be explained by neuronal plasticity resulting in downregulation of ChAT following general reduction of enteric neurons. Adaptive changes in the proportions of the different neuronal populations occur as a result of intestinal dysfunction following injury [49, 50]. To date, similar studies on enteric innervation under cachexia are rare. Therefore, we have examined innervation in other pathological conditions for interpretation of the present results. Neuronal atrophy, including in ChAT-IR neurons as a result of oxidative injury, has been reported in other disease states [38, 47]. In human pancreatic cancer a reduction in pancreatic cholinergic innervation was reported, associated with visceral neuropathy due to invasion by cancer cells [51]. In the present study, submucous CHAT-containing neurons could not be assessed because of the lack of appropriate samples.

Hypertrophy of remaining VIP-containing submucous neurons and myenteric varicosities was associated with higher expression of VIP in cancer cachexia. Plastic neuronal changes are common, especially for VIP-IR neurons and nerve fibers in the plexus under damage conditions such as intestinal inflammatory diseases in patients, and in experimental models [50, 52]. Therefore, this plasticity may be linked to the neuroprotective effect of VIP arising from its antioxidant, anti-inflammatory, and mucosa protective effects [24, 25, 52]. However, defects in axonal transport of VIP, common in neurodegenerative processes [52], may be present in cachexia and lead to accumulation of this neuropeptide in varicosity. A study has shown an increase in the density of VIP-expressing myenteric fibers in experimental colorectal carcinoma, highlighting their cytotoxic effect against tumor cells, but with no accompanying changes in neuronal density [19]. Other studies have reported a decrease in VIP expression by nerve fibers, while VIPergic neurons showed no changes in density in human colon cancer [18]. Changes in VIP-expressing neural elements may alter motility and absorption in the small intestine [14, 18, 25] and result in intestinal obstruction, a common and severe complication of inflammatory and neoplastic diseases in the gastrointestinal tract [50].

CGRP neuropeptide participates in sensory reflexes of the intestinal wall [14, 17]. CGRP-containing nerve fibers arise from myenteric and submucous neurons and from afferent sensory neurons, whose cell bodies are extrinsically located [23, 53]. In addition to acting as a vasodilator [14], CGRP also possesses anti-inflammatory [54] and antioxidant properties [55]. As for VIP-IR varicosities, hypertrophic CGRP-containing varicosities were associated with higher CGRP expression, implying an adaptive response to systemic effects of cachexia. In this study, the analysis methods do not allow differentiation between intrinsic and extrinsic CGRP-IR nerve fibers. Contrary to our findings, a study revealed a decrease in CGRP-IR neurons and nerve fibers in human carcinoma, and tumoral invasion was indicated to be the cause of this neurodegeneration [17]. On the other hand, another study has suggested that the tumor promotes CGRP overproduction, working as a growth factor that stimulates angiogenesis and lymphangiogenesis, favoring cancer progression [56]. In addition, upregulation of CGRP expression has been observed in tumor-bearing mice in which blocking of CGRP production was associated with the reduction of tumor growth following denervation [57].

### l-Glutamine Effects on Enteric Neurons

Most of the effects of l-glutamine on nerve cells are due to its conversion into glutathione which exerts an antioxidant effect through various mechanisms [8, 33, 58], comprising modulation in calcium homeostasis, apoptosis, and the intestinal immune system [10, 41]. l-glutamine also has been shown to have antioxidant effects [59] through influencing mechanisms of GSH synthesis and recycling, improving the availability of GSH [60, 61]. In addition, several studies have highlighted l-glutamine neuroprotective effects [59, 62], even in the enteric nervous system [31], preventing the shrinkage and myenteric neuronal loss in several intestinal segments [31, 32, 35]. We observed a protective effect on neuronal density only in the ileal segment. Similar results regarding its lack of effect in the jejunum were described for both the myenteric and submucosal plexuses in a recent study involving experimental DM [33], while another study described the neuroprotective effect of l-glutamine in the ileum [31]. In another report, the authors described the preservation of myenteric neurons in the duodenum, although not in the cecum after supplementation with l-glutamine [32]. These observations suggest that each intestinal segment utilizes distinct intrinsic mechanisms when subjected with the same treatment under conditions of injury.

l-glutamine supplementation did not provide preventive effect on submucosal neuronal densities (HuC / D-IR and VIP-IR). This lack of effect in this plexus has been previously reported during the development of the diabetic enteric neuropathy [33]. Another recent study also revealed that l-glutamine exerted a differential neuroprotective effect in experimental diabetes that depend on the type of neurotransmitter analyzed [63].

The preventive effects of l-glutamine on hypertrophy of the VIP-containing neurons and fibers have been previously reported [25, 33]. In addition the upregulation of VIP expression was also prevented. Therefore, l-glutamine is expected to exert neuroprotective effects during the development of cancer-related neuropathy and, with greater circulating antioxidant availability [58, 59, 61], enteric neurons will not need to express large amounts of VIP, thus maintaining normal neuron and varicosity size.

l-glutamine prevented the changes in size of CGRP-IR varicosities in this study. Although there is no study available on this issue, it is likely that this effect may occur also through mitigation of CGRP release by glutathione [64]. However, there have been isolated cases in which an unexpected abnormal neuron hypertrophy and a slight increase in the size of VIP- and CGRP-containing varicosities were noted when the diets of healthy or cachexia rats were supplemented with l-glutamine. Previous studies have suggested a possible toxic effect induced by excessive release of l-glutamate, produced as a consequence of excess l-glutamine or glutathione due to metabolic defects, leading to cellular edema and resulting in varicose hypertrophy [8, 33]. As these changes were not followed by overexpression of the peptides, it also suggests an causal effect that may even reflect an improvement in VIP and CGRP signaling in neuroregulatory functions [23, 25], since l-glutamine also has a trophic effect on the mucosa and submucosa [65].

## Conclusion

In conclusion, we determined that cancer cachexia severely affects the ENS in the jejunum and ileum to various degrees. However, there is a neuroplastic response to this injury, highlighted by the higher neuropeptide expression. l-glutamine was beneficial both for physiological parameters as well as for preventing neuronal density reduction, specifically in the myenteric plexus of the ileum. Moreover, l-glutamine prevented morphometric changes (atrophy and hypertrophy) in neurons and nerve fibers in the myenteric and submucosal plexuses of the segments investigated. As proposed in this and other studies, we suggest that l-glutamine has protective effects on the ENS under cancer-related cachexia.

## Supporting Information

S1 Fig Representative images of myenteric neurons of ileum from experimental groups: control (C); control supplemented with 2% L-glutamine (CG); Walker-256 tumor (TW); and Walker-256 tumor supplemented with 2% L-glutamine (TWG). (a-d) ChAT-IR subpopulation (red). Magnification images (× 6) of the isolated ChAT-IR (a'-d') myenteric neurons are shown on the top left of each image. Scale Bar 50 μm.
(TIF)

S2 Fig Representative images of myenteric neurons of jejunum from experimental groups: control (C); control supplemented with 2% L-glutamine (CG); Walker-256 tumor (TW); and Walker-256 tumor supplemented with 2% L-glutamine (TWG). (a-d) ChAT-IR subpopulation (red). Magnification images (× 6) of the isolated ChAT-IR (a'-d') myenteric neurons are shown on the top left of each image. Scale Bar 50 μm.
(TIF)

S1 Table Neuronal density (uncorrected) of myenteric neurons (neurons/cm^2^) in the jejunum and ileum. **Experimental groups: control (C); control supplemented with 2% L-glutamine (CG); Walker-256 tumor (TW); and Walker-256 tumor supplemented with 2% L-glutamine (TWG). Uncorrected HuC/D-IR population (neurons/cm^2^), CHAT-immunoreactive subpopulation (neurons/cm^2^).** The results are expressed as mean ± SEM (n = 8). Means followed by different letters in the same row are significantly different (p < 0.05) according to ANOVA (two-way) followed Tukey's post hoc test.
(PDF)

S2 Table Neuronal density (uncorrected) of submucous neurons (neurons/cm^2^) in the jejunum and ileum. **Experimental groups: control (C); control supplemented with 2% L-glutamine (CG); Walker-256 tumor (TW); and Walker-256 tumor supplemented with 2% L-glutamine (TWG). Uncorrected HuC/D-IR population (neurons/cm^2^) and VIP-immunoreactive subpopulation (neurons/cm^2^) densities. The results are expressed as mean ± SEM (n = 8).** Means followed by different letters in the same row are significantly different (p < 0.05) according to ANOVA (two-way) followed Tukey's post hoc test.
(PDF)

## References

[1] Argiles JM, Busquets S, Stemmler B, Lopez-Soriano FJ. Cancer cachexia: understanding the molecular basis. Nature reviews Cancer. 2014;14(11):754–62. Epub 2014/10/08. 10.1038/nrc3829 . 25291291

[2] Fearon K, Strasser F, Anker SD, Bosaeus I, Bruera E, Fainsinger RL, et al Definition and classification of cancer cachexia: an international consensus. The Lancet Oncology. 2011;12(5):489–95. Epub 2011/02/08. 10.1016/S1470-2045(10)70218-7 . 21296615

[3] Mantovani G, Madeddu C, Maccio A. Cachexia and oxidative stress in cancer: an innovative therapeutic management. Current pharmaceutical design. 2012;18(31):4813–8. Epub 2012/05/29. . 22632861 10.2174/138161212803216889

[4] Fearon KC, Glass DJ, Guttridge DC. Cancer cachexia: mediators, signaling, and metabolic pathways. Cell metabolism. 2012;16(2):153–66. Epub 2012/07/17. 10.1016/j.cmet.2012.06.011 . 22795476

[5] Guarnier FA, Cecchini AL, Suzukawa AA, Maragno AL, Simao AN, Gomes MD, et al Time course of skeletal muscle loss and oxidative stress in rats with Walker 256 solid tumor. Muscle & nerve. 2010;42(6):950–8. Epub 2010/11/26. 10.1002/mus.21798 . 21104869

[6] Gandhi S, Abramov AY. Mechanism of oxidative stress in neurodegeneration. Oxidative medicine and cellular longevity. 2012;2012:428010 Epub 2012/06/12. 10.1155/2012/428010 22685618 PMC3362933

[7] Halliwell B. Oxidative stress and neurodegeneration: where are we now? Journal of neurochemistry. 2006;97(6):1634–58. Epub 2006/06/30. 10.1111/j.1471-4159.2006.03907.x . 16805774

[8] Aoyama K, Watabe M, Nakaki T. Regulation of neuronal glutathione synthesis. Journal of pharmacological sciences. 2008;108(3):227–38. Epub 2008/11/15. . 19008644 10.1254/jphs.08r01cr

[9] Zhang F, Wang X, Wang W, Li N, Li J. Glutamine reduces TNF-alpha by enhancing glutathione synthesis in lipopolysaccharide-stimulated alveolar epithelial cells of rats. Inflammation. 2008;31(5):344–50. Epub 2008/09/23. 10.1007/s10753-008-9084-0 . 18807160

[10] Michalak KP, Mackowska-Kedziora A, Sobolewski B, Wozniak P. Key Roles of Glutamine Pathways in Reprogramming the Cancer Metabolism. Oxidative medicine and cellular longevity. 2015;2015:964321 Epub 2015/11/20. 10.1155/2015/964321 26583064 PMC4637129

[11] Ramos Lima MM, de Mello MA, Curi R. Walker 256 tumour growth causes marked changes of glutamine metabolism in rat small intestine. Cell biochemistry and function. 2002;20(2):107–13. Epub 2002/04/30. 10.1002/cbf.961 . 11979506

[12] DiBaise JK, Quigley EM. Tumor-related dysmotility: gastrointestinal dysmotility syndromes associated with tumors. Digestive diseases and sciences. 1998;43(7):1369–401. Epub 1998/08/05. . 9690371 10.1023/a:1018853106696

[13] Godlewski J. Morphological changes in the enteric nervous system caused by carcinoma of the human large intestine. Folia histochemica et cytobiologica / Polish Academy of Sciences, Polish Histochemical and Cytochemical Society. 2010;48(1):157–62. Epub 2010/06/10. 10.2478/v10042-010-0029-8 . 20529833

[14] Furness JB. The enteric nervous system. Malden, Mass.: Blackwell Pub.; 2006 xiii, 274 p. p.

[15] Mann PT, Furness JB, Southwell BR. Choline acetyltransferase immunoreactivity of putative intrinsic primary afferent neurons in the rat ileum. Cell and tissue research. 1999;297(2):241–8. Epub 1999/09/02. . 10470494 10.1007/s004410051352

[16] Godlewski J, Pidsudko Z. Characteristic of galaninergic components of the enteric nervous system in the cancer invasion of human large intestine. Annals of anatomy = Anatomischer Anzeiger: official organ of the Anatomische Gesellschaft. 2012;194(4):368–72. Epub 2012/01/10. 10.1016/j.aanat.2011.11.009 . 22226150

[17] Godlewski J, Kaleczyc J. Somatostatin, substance P and calcitonin gene-related peptide-positive intramural nerve structures of the human large intestine affected by carcinoma. Folia histochemica et cytobiologica / Polish Academy of Sciences, Polish Histochemical and Cytochemical Society. 2010;48(3):475–83. Epub 2010/11/13. 10.2478/v10042-010-0079-y . 21071356

[18] Godlewski J, Lakomy IM. Changes in vasoactive intestinal peptide, pituitary adenylate cyclase-activating polypeptide and neuropeptide Y-ergic structures of the enteric nervous system in the carcinoma of the human large intestine. Folia histochemica et cytobiologica / Polish Academy of Sciences, Polish Histochemical and Cytochemical Society. 2010;48(2):208–16. Epub 2010/08/03. 10.2478/v10042-010-0052-9 . 20675276

[19] Sitohy B, El-Salhy M. Changes in the colonic enteric nervous system in rats with chemically induced colon dysplasia and carcinoma. Acta Oncol. 2002;41(6):543–9. Epub 2003/01/28. . 12546527 10.1080/02841860214957

[20] Levine BA, Kaplan BJ. Polyps and polypoid lesions of the jejunum and ileum. Clinical aspects. Surgical oncology clinics of North America. 1996;5(3):609–19. Epub 1996/07/01. . 8829322

[21] Sitohy B, El-Salhy M. Colonic endocrine cells in rats with chemically induced colon carcinoma. Histology and histopathology. 2001;16(3):833–8. Epub 2001/08/21. . 11510974 10.14670/HH-16.833

[22] El-Salhy M, Mahdavi J, Norrgard O. Colonic endocrine cells in patients with carcinoma of the colon. European journal of gastroenterology & hepatology. 1998;10(6):517–22. Epub 1998/12/17. . 9855070 10.1097/00042737-199806000-00015

[23] Chiocchetti R, Grandis A, Bombardi C, Lucchi ML, Dal Lago DT, Bortolami R, et al Extrinsic and intrinsic sources of calcitonin gene-related peptide immunoreactivity in the lamb ileum: a morphometric and neurochemical investigation. Cell and tissue research. 2006;323(2):183–96. Epub 2005/10/18. 10.1007/s00441-005-0075-2 . 16228232

[24] Tuncel N, Korkmaz OT, Tekin N, Sener E, Akyuz F, Inal M. Antioxidant and anti-apoptotic activity of vasoactive intestinal peptide (VIP) against 6-hydroxy dopamine toxicity in the rat corpus striatum. Journal of molecular neuroscience: MN. 2012;46(1):51–7. Epub 2011/08/19. 10.1007/s12031-011-9618-z . 21850490

[25] Alves EP, Alves AM, Pereira RV, de Miranda Neto MH, Zanoni JN. Immunohistochemical study of vasoactive intestinal peptide (VIP) enteric neurons in diabetic rats supplemented with L-glutamine. Nutritional neuroscience. 2010/02/06 ed2010. p. 43–51. 20132654 10.1179/147683010X12611460763841

[26] Guaitani A, Recchia M, Carli M, Rocchetti M, Bartosek I, Garattini S. Walker carcinoma 256: a model for studies on tumor-induced anorexia and cachexia. Oncology. 1982;39(3):173–8. Epub 1982/01/01. . 6952138 10.1159/000225631

[27] Freitas JJ, Pompeia C, Miyasaka CK, Curi R. Walker-256 tumor growth causes oxidative stress in rat brain. Journal of neurochemistry. 2001;77(2):655–63. Epub 2001/04/12. . 11299328 10.1046/j.1471-4159.2001.00280.x

[28] Arias-Rotunno LR. Perfil do estresse oxidativo nas hemácias e plasma de animais com caquexia induzida por tumor de Walker-256 [Dissertation (Masters in Experimental Pathology)]. Universidade Estadual de Londrina: Universidade Estadual de Londrina; 2007.

[29] Rebeca R, Bracht L, Noleto GR, Martinez GR, Cadena SM, Carnieri EG, et al Production of cachexia mediators by Walker 256 cells from ascitic tumors. Cell biochemistry and function. 2008;26(6):731–8. Epub 2008/07/23. 10.1002/cbf.1497 . 18646274

[30] Fracaro L, Frez FC, Silva BC, Vicentini GE, de Souza SR, Martins HA, et al Walker 256 tumor-bearing rats demonstrate altered interstitial cells of Cajal. Effects on ICC in the Walker 256 tumor model. Neurogastroenterology and motility: the official journal of the European Gastrointestinal Motility Society. 2015 Epub 2015/11/04. 10.1111/nmo.12702 . 26526599 PMC4688090

[31] Pereira RV, Tronchini EA, Tashima CM, Alves EP, Lima MM, Zanoni JN. L-glutamine supplementation prevents myenteric neuron loss and has gliatrophic effects in the ileum of diabetic rats. Digestive diseases and sciences. 2011;56(12):3507–16. Epub 2011/06/29. 10.1007/s10620-011-1806-8 . 21710226

[32] Zanoni JN, Tronchini EA, Moure SA, Souza ID. Effects of L-glutamine supplementation on the myenteric neurons from the duodenum and cecum of diabetic rats. Arquivos de gastroenterologia. 2011;48(1):66–71. Epub 2011/05/04. . 21537546 10.1590/s0004-28032011000100014

[33] Hermes-Uliana C, Panizzon CP, Trevizan AR, Sehaber CC, Ramalho FV, Martins HA, et al Is L-glutathione more effective than L-glutamine in preventing enteric diabetic neuropathy? Digestive diseases and sciences. 2014;59(5):937–48. Epub 2013/12/29. 10.1007/s10620-013-2993-2 . 24370785

[34] Iagher F, de Brito Belo SR, Souza WM, Nunes JR, Naliwaiko K, Sassaki GL, et al Antitumor and anti-cachectic effects of shark liver oil and fish oil: comparison between independent or associative chronic supplementation in Walker 256 tumor-bearing rats. Lipids in health and disease. 2013;12:146 Epub 2013/10/18. 10.1186/1476-511X-12-146 24131597 PMC4015826

[35] Tashima CM, Tronchini EA, Pereira RV, Bazotte RB, Zanoni JN. Diabetic rats supplemented with L-glutamine: a study of immunoreactive myosin-V myenteric neurons and the proximal colonic mucosa. Digestive diseases and sciences. 2007;52(5):1233–41. Epub 2007/03/30. 10.1007/s10620-006-9564-8 . 17393333

[36] Costa M, Buffa R, Furness JB, Solcia E. Immunohistochemical localization of polypeptides in peripheral autonomic nerves using whole mount preparations. Histochemistry. 1980;65(2):157–65. Epub 1980/02/01. . 6987197 10.1007/BF00493164

[37] Bradford MM. A rapid and sensitive method for the quantitation of microgram quantities of protein utilizing the principle of protein-dye binding. Analytical biochemistry. 1976;72:248–54. Epub 1976/05/07. . 942051 10.1016/0003-2697(76)90527-3

[38] Soares A, Beraldi EJ, Ferreira PE, Bazotte RB, Buttow NC. Intestinal and neuronal myenteric adaptations in the small intestine induced by a high-fat diet in mice. BMC gastroenterology. 2015;15:3 Epub 2015/01/23. 10.1186/s12876-015-0228-z 25609418 PMC4316644

[39] Cowen T, Johnson RJ, Soubeyre V, Santer RM. Restricted diet rescues rat enteric motor neurones from age related cell death. Gut. 2000;47(5):653–60. Epub 2000/10/18. 11034581 10.1136/gut.47.5.653 PMC1728112

[40] Phillips RJ, Powley TL. Innervation of the gastrointestinal tract: patterns of aging. Autonomic neuroscience: basic & clinical. 2007;136(1–2):1–19. Epub 2007/06/01. 10.1016/j.autneu.2007.04.005 17537681 PMC2045700

[41] Kuhn KS, Muscaritoli M, Wischmeyer P, Stehle P. Glutamine as indispensable nutrient in oncology: experimental and clinical evidence. European journal of nutrition. 2010;49(4):197–210. Epub 2009/11/26. 10.1007/s00394-009-0082-2 . 19936817

[42] Souba WW, Strebel FR, Bull JM, Copeland EM, Teagtmeyer H, Cleary K. Interorgan glutamine metabolism in the tumor-bearing rat. The Journal of surgical research. 1988;44(6):720–6. Epub 1988/06/01. . 3379949 10.1016/0022-4804(88)90106-0

[43] Johnson RJ, Schemann M, Santer RM, Cowen T. The effects of age on the overall population and on sub-populations of myenteric neurons in the rat small intestine. Journal of anatomy. 1998;192 (Pt 4):479–88. Epub 1998/09/02. 9723975 10.1046/j.1469-7580.1998.19240479.x PMC1467802

[44] Gamage PP, Ranson RN, Patel BA, Yeoman MS, Saffrey MJ. Myenteric neuron numbers are maintained in aging mouse distal colon. Neurogastroenterology and motility: the official journal of the European Gastrointestinal Motility Society. 2013;25(7):e495–e505. Epub 2013/03/23. 10.1111/nmo.12114 . 23517051

[45] Machado AP, Costa Rosa LF, Seelaender MC. Adipose tissue in Walker 256 tumour-induced cachexia: possible association between decreased leptin concentration and mononuclear cell infiltration. Cell and tissue research. 2004;318(3):503–14. Epub 2004/10/19. 10.1007/s00441-004-0987-2 . 15490241

[46] Fenner FL, Guarnier FA, Bernardes SS, Ramalho LN, Cecchini R, Cecchini AL. Increased nitric oxide levels in cerebellum of cachectic rats with Walker 256 solid tumor. Folia neuropathologica / Association of Polish Neuropathologists and Medical Research Centre, Polish Academy of Sciences. 2015;53(2):139–46. Epub 2015/07/29. . 26216116 10.5114/fn.2015.52410

[47] Paulino AS, Palombit K, Cavriani G, Tavares-de-Lima W, Mizuno MS, Marosti AR, et al Effects of ischemia and reperfusion on P2X2 receptor expressing neurons of the rat ileum enteric nervous system. Digestive diseases and sciences. 2011;56(8):2262–75. Epub 2011/03/17. 10.1007/s10620-011-1588-z . 21409380

[48] Kurnik M, Gil K, Gajda M, Thor P, Bugajski A. Neuropathic alterations of the myenteric plexus neurons following subacute intraperitoneal administration of salsolinol. Folia histochemica et cytobiologica / Polish Academy of Sciences, Polish Histochemical and Cytochemical Society. 2015;53(1):49–61. Epub 2015/03/31. 10.5603/FHC.a2015.0010 . 25815627

[49] Ekelund KM, Ekblad E. Structural, neuronal, and functional adaptive changes in atrophic rat ileum. Gut. 1999;45(2):236–45. Epub 1999/07/14. 10403736 10.1136/gut.45.2.236 PMC1727608

[50] Ekblad E, Sjuve R, Arner A, Sundler F. Enteric neuronal plasticity and a reduced number of interstitial cells of Cajal in hypertrophic rat ileum. Gut. 1998;42(6):836–44. Epub 1998/08/06. 9691923 10.1136/gut.42.6.836 PMC1727150

[51] Ceyhan GO, Demir IE, Rauch U, Bergmann F, Muller MW, Buchler MW, et al Pancreatic neuropathy results in "neural remodeling" and altered pancreatic innervation in chronic pancreatitis and pancreatic cancer. The American journal of gastroenterology. 2009;104(10):2555–65. Epub 2009/07/02. 10.1038/ajg.2009.380 . 19568227

[52] Ekblad E, Bauer AJ. Role of vasoactive intestinal peptide and inflammatory mediators in enteric neuronal plasticity. Neurogastroenterology and motility: the official journal of the European Gastrointestinal Motility Society. 2004;16 Suppl 1:123–8. Epub 2004/04/07. 10.1111/j.1743-3150.2004.00487.x . 15066017

[53] Rasmussen TN, Schmidt P, Poulsen SS, Holst JJ. Localisation and neural control of the release of calcitonin gene-related peptide (CGRP) from the isolated perfused porcine ileum. Regulatory peptides. 2001;98(3):137–43. Epub 2001/03/07. . 11231043 10.1016/s0167-0115(00)00242-1

[54] Holzmann B. Modulation of immune responses by the neuropeptide CGRP. Amino acids. 2013;45(1):1–7. Epub 2011/11/25. 10.1007/s00726-011-1161-2 . 22113645

[55] Wu Y, Hao GM, He J, Lv TT, Wang HL, Mao YQ, et al Lentivirus mediated over expression of CGRP inhibited oxidative stress in Schwann cell line. Neuroscience letters. 2015;598:52–8. Epub 2015/05/12. 10.1016/j.neulet.2015.05.009 . 25960317

[56] Hay DL, Walker CS, Poyner DR. Adrenomedullin and calcitonin gene-related peptide receptors in endocrine-related cancers: opportunities and challenges. Endocrine-related cancer. 2011;18(1):C1–14. Epub 2010/11/06. 10.1677/ERC-10-0244 . 21051558

[57] Toda M, Suzuki T, Hosono K, Hayashi I, Hashiba S, Onuma Y, et al Neuronal system-dependent facilitation of tumor angiogenesis and tumor growth by calcitonin gene-related peptide. Proceedings of the National Academy of Sciences of the United States of America. 2008;105(36):13550–5. Epub 2008/09/02. 10.1073/pnas.0800767105 18757746 PMC2527353

[58] Amores-Sanchez MI, Medina MA. Glutamine, as a precursor of glutathione, and oxidative stress. Molecular genetics and metabolism. 1999;67(2):100–5. Epub 1999/06/05. . 10356308 10.1006/mgme.1999.2857

[59] Chen J, Herrup K. Glutamine acts as a neuroprotectant against DNA damage, beta-amyloid and H2O2-induced stress. PloS one. 2012;7(3):e33177 Epub 2012/03/14. 10.1371/journal.pone.0033177 22413000 PMC3297635

[60] Shin EJ, Nam Y, Tu TT, Lim YK, Wie MB, Kim DJ, et al Protein kinase Cdelta mediates trimethyltin-induced neurotoxicity in mice in vivo via inhibition of glutathione defense mechanism. Archives of toxicology. 2015 Epub 2015/04/22. 10.1007/s00204-015-1516-7 . 25895139

[61] Wang AL, Niu Q, Shi N, Wang J, Jia XF, Lian HF, et al Glutamine ameliorates intestinal ischemia-reperfusion Injury in rats by activating the Nrf2/Are signaling pathway. International journal of clinical and experimental pathology. 2015;8(7):7896–904. Epub 2015/09/05. 26339354 PMC4555682

[62] Amara S. Oral glutamine for the prevention of chemotherapy-induced peripheral neuropathy. The Annals of pharmacotherapy. 2008;42(10):1481–5. Epub 2008/08/14. 10.1345/aph.1L179 . 18698011

[63] Pereira RV, Linden DR, Miranda-Neto MH, Zanoni JN. Differential effects in CGRPergic, nitrergic, and VIPergic myenteric innervation in diabetic rats supplemented with 2% L-glutamine. Anais da Academia Brasileira de Ciencias. 2016;88 Suppl 1:609–22. Epub 2016/05/05. 10.1590/0001-3765201620150228 . 27142540

[64] Materazzi S, Fusi C, Benemei S, Pedretti P, Patacchini R, Nilius B, et al TRPA1 and TRPV4 mediate paclitaxel-induced peripheral neuropathy in mice via a glutathione-sensitive mechanism. Pflugers Archiv: European journal of physiology. 2012;463(4):561–9. Epub 2012/01/20. 10.1007/s00424-011-1071-x . 22258694

[65] Rosa CV, Azevedo SC, Bazotte RB, Peralta RM, Buttow NC, Pedrosa MM, et al Supplementation with L-Glutamine and L-Alanyl-L-Glutamine Changes Biochemical Parameters and Jejunum Morphophysiology in Type 1 Diabetic Wistar Rats. PloS one. 2015;10(12):e0143005 Epub 2015/12/15. 10.1371/journal.pone.0143005 26659064 PMC4681705

