# Supplementary figures and images for: Experimental Cancer Cachexia Changes Neuron Numbers and Peptide Levels in the Intestine: Partial Protective Effects after Dietary Supplementation with L-Glutamine
Source: PLoS One. 2016 Sep 16;11(9):e0162998. doi: 10.1371/journal.pone.0162998 (PMC5026352; doi:10.1371/journal.pone.0162998)

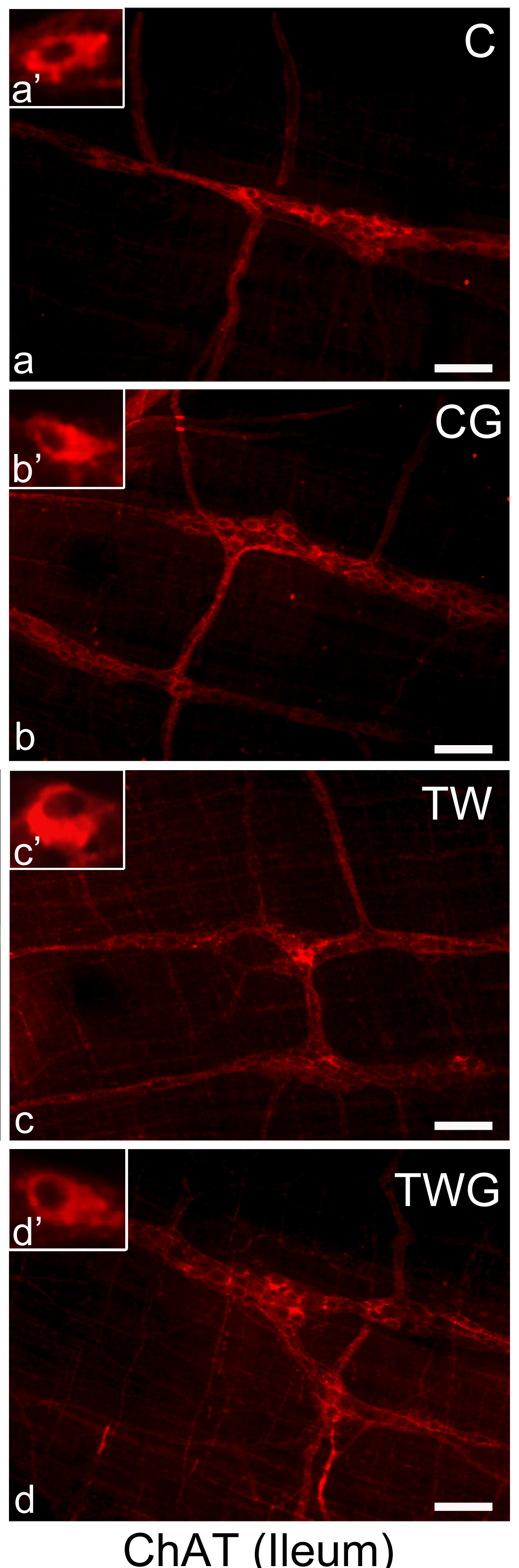

Supplement: S1 Fig — (a-d) ChAT-IR subpopulation (red). Magnification images (× 6) of the isolated ChAT-IR (a'-d') myenteric neurons are shown on the top left of each image. Scale Bar 50 μm. (TIF) [file pone.0162998.s001.tif]

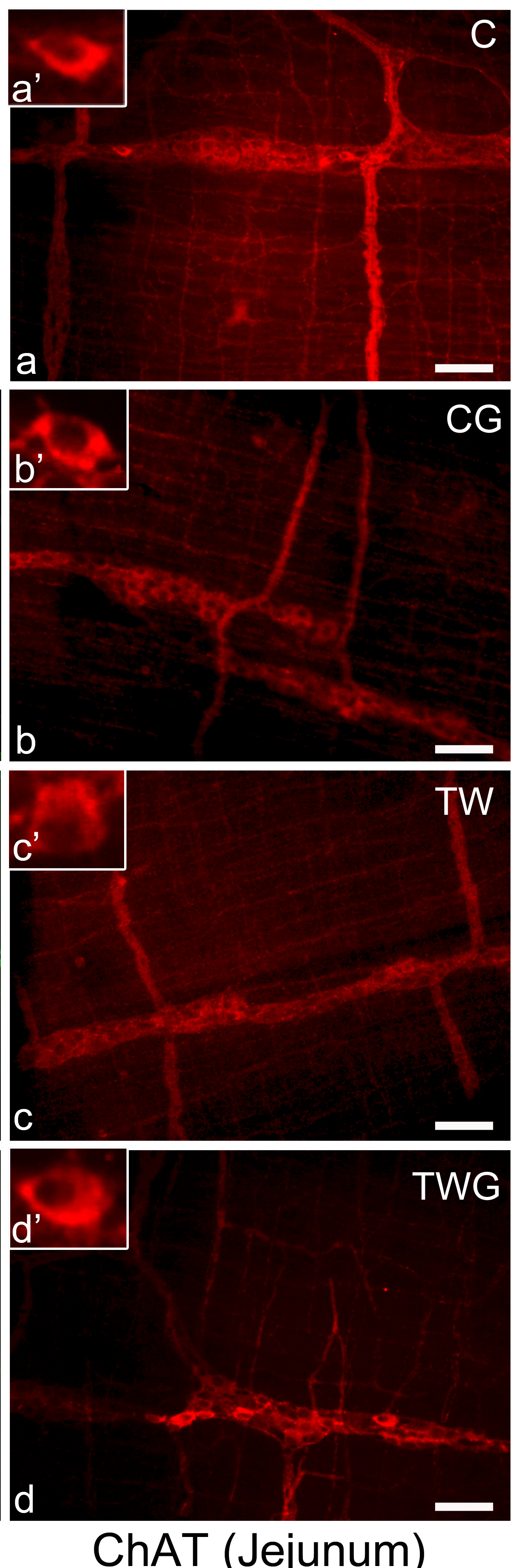

Supplement: S2 Fig — (a-d) ChAT-IR subpopulation (red). Magnification images (× 6) of the isolated ChAT-IR (a'-d') myenteric neurons are shown on the top left of each image. Scale Bar 50 μm. (TIF) [file pone.0162998.s002.tif]
