# Supplementary material for: Experimental Cancer Cachexia Changes Neuron Numbers and Peptide Levels in the Intestine: Partial Protective Effects after Dietary Supplementation with L-Glutamine
Source: PLoS One. 2016 Sep 16;11(9):e0162998. doi: 10.1371/journal.pone.0162998 (PMC5026352; doi:10.1371/journal.pone.0162998)
Supplement: S1 Table — Experimental groups: control (C); control supplemented with 2% L-glutamine (CG); Walker-256 tumor (TW); and Walker-256 tumor supplemented with 2% L-glutamine (TWG). Uncorrected HuC/D-IR population (neurons/cm2), CHAT-immunoreactive subpopulation (neurons/cm2). The results are expressed as mean ± SEM (n = 8). Means followed by different letters in the same row are significantly different (p < 0.05) according to ANOVA (two-way) followed Tukey's post hoc test. (PDF) [file pone.0162998.s003.pdf]

S1 Table.

		Experimental Groups			
		**C**	**CG**	**TW**	**TWG**
**Jejunum**	HuC/D	14986.3 ± 499.9[a]	16734.90 ± 406.1[b]	15383.1 ± 472.8[a]	15283.6 ± 389.2[a]
	CHAT	11233.9 ± 483.4[a,b]	12518.3 ± 232.5[b]	10449.4 ± 315.3[a,c]	11025.8 ± 494.8[a,b]
**Ileum**	HuC/D	20843.9 ± 835.4[a]	21920.4 ± 471.9[a]	21561.1 ± 846.9[a]	23485.7 ± 531.4[a]
	CHAT	15270.9 ± 606.1[a]	15900.5 ± 516.8[a]	13151.5 ± 415.4[a]	15213.8 ± 574.8[a]

The results are expressed as mean ± SEM (n = 8).

Means followed by different letters in the same row are significantly different ($p < 0.05$) according to ANOVA (two-way) followed Tukey's post hoc test.
