# Supplementary material for: Experimental Cancer Cachexia Changes Neuron Numbers and Peptide Levels in the Intestine: Partial Protective Effects after Dietary Supplementation with L-Glutamine
Source: PLoS One. 2016 Sep 16;11(9):e0162998. doi: 10.1371/journal.pone.0162998 (PMC5026352; doi:10.1371/journal.pone.0162998)
Supplement: S2 Table — Experimental groups: control (C); control supplemented with 2% L-glutamine (CG); Walker-256 tumor (TW); and Walker-256 tumor supplemented with 2% L-glutamine (TWG). Uncorrected HuC/D-IR population (neurons/cm2) and VIP-immunoreactive subpopulation (neurons/cm2) densities. The results are expressed as mean ± SEM (n = 8). Means followed by different letters in the same row are significantly different (p < 0.05) according to ANOVA (two-way) followed Tukey's post hoc test. (PDF) [file pone.0162998.s004.pdf]

S2 Table.

		Experimental Groups			
		**C**	**CG**	**TW**	**TWG**
**Jejunum**	HuC/D	9451.1 ± 154.3[a]	10217.3 ± 257.7[b]	9698.1 ± 234.2[a]	9321.8 ± 167.4[a]
	VIP	4234.9 ± 121.1[a]	4714.1 ± 89.1[b]	5206.3 ± 118.3[c]	4818.2 ± 137.3[b]
**Ileum**	HuC/D	8738.6 ± 268.5[a,c]	8976.4 ± 222.3[a]	9809.1 ± 220.3[b]	7837.4 ± 207.1[c]
	VIP	3985.6 ± 78.2[a,b]	4125.1 ± 90.7[a]	4293.3 ± 121.4[a]	3658.5 ± 108.9[b]

Means followed by different letters in the same row are significantly different ($p < 0.05$) according to ANOVA (two-way) followed Tukey's post hoc test
